# A Theoretical and Practical Treatise on Wounds by Military Weapons; Composed from the Clinical Lectures of M. le Baron Dupuytren, and Published, under His Inspection

**Published:** 1834-10-01

**Authors:** 


*Traité, Théorique et Pratique, des Blessures par Armes de Guerre; rédigé d'après les Leçons Cliniques de* M. le Baron Dupuytren, Chirurgien en Chef de l'Hôtel-Dieu; *et publié sous sa direction par* MM. les Docteurs A. Paillard et Marx. Tome 1er.—Paris, 1834. 8vo. pp. 522.

*A Theoretical and Practical Treatise on Wounds by Military Weapons; composed from the Clinical Lectures of* M. le Baron Dupuytren, *and published, under his inspection,* by Doctors Paillard and Marx.

However politicians may differ as to the French revolution of 1830, there is perhaps one point of view, from which it may be contemplated as a source of advantage to mankind. The three days of July afforded to Baron Dupuytren, whose skill as a surgeon is perhaps unequalled throughout Europe, a field of experience in wounds from weapons, at once most extensive and most favourably disposed for observation. "Let history," says the Baron, "relate the causes that led to this solemn catastrophe, and what have been its effects upon the country; let us, as surgeons, devoted no less by duty than by inclination to the relief of human suffering, confine ourselves to the study of that which was done, in the midst of the combat, to alleviate the tortures of its victims." This celebrated surgeon, suddenly called, as the head of the medical staff of Paris, to assume a most responsible situation in the military department of our art, though attached to a civil hospital, did not come to the task wholly unprepared by previous experience. Under the tuition of Boyer, at that time the first surgeon in France, he studied at La Charité during the contest of the 13 Vendémiaire; and again, in the years 1814 and 1815, the engagements with the allies, under the walls of Paris, gave occasion to the exercise of his skill on the field of battle. So great indeed was his zeal on the former of these occasions, that, having laboured incessantly from five o'clock in the morning till half-past five in the evening, he did not commence his retreat till a cannon-ball had carried away the legs of one of his attendants, and till the closure of the gates of the capital had nearly left him to the mercy of the enemy. At this period, five hundred cases came under his personal observation; and the skirmish of General Excelmans with the Prussians, near Versailles, in the year 1815, afforded him still further opportunities. The riots at St. Denis, in 1827, served to refresh his memory on the subject, prior to the grand struggle of 1830, when upwards of four hundred severe cases, besides an immense number of slighter injuries, were committed to his charge. Thus prepared by previous experience, no less than by a very superior knowledge of general surgery, acquired by a superintendence of one of the largest civil hospitals in Europe, Baron Dupuytren was extraordinarily fitted for improving the opportunities afforded to him. Neither were these opportunities a whit inferior to his capacity: his patients were numerous, some under the excitation of victory, others under the depression of defeat; some, military men, in high and vigorous health, others artizans, with constitutions weakened by residence in a metropolitan city. The wounds were produced by all kinds of weapons, and not confined to those ordinarily employed in regular warfare. To all these advantages may be added, that of his appointment as surgeon to the convalescent hospital of St. Cloud, by which he was enabled to keep the cases under his eye until their cures were completed. The record of this experience is now before us, published, under his superintendence, by his pupils, Drs. Paillard and Marx, and, when concluded, will pay the debt which he had contracted to science, and will form a most admirable digest of military surgery.

At the outset of the work is a condensed account of the various kinds of weapons by which wounds may be produced, from the needle to the cannon-ball; but, though this may render the work more complete, it is evidently unfitted for comment in a Medical Review: suffice it to say, that they are arranged according to the nature of the wounds which they inflict, being divided into puncturing, cutting, puncturing and cutting, tearing, dragging, bruising, and crushing arms; air and steam guns, portable firearms, cannons, mines, rockets; bullets, balls, and other projectiles, and the different species of exploding powders.

In the section on *Punctured Wounds through soft Parts,* a case is related, of a man who attempted to commit suicide by puncturing the heart with a pointed bodkin. He was admitted into the Hôtel-Dieu, where it was observed that the wounds were long and collapsed, though made with a rounded instrument. This led the Baron to institute a series of experiments on the dead body, as to the nature and form of punctured wounds. The experiments were performed with a conical needle, about three inches long, and three and a quarter lines in diameter at its largest part, and the following were the results which he obtained:

"1st. The wounds produced by this instrument were never round, but long, with the edges even and collapsed.

"2d. The length of the wounds in the skin corresponded with the depth to which the instrument was introduced.

"3d. If, at any part of the body, the edges of the wound remained open, stretching the skin would readily make them collapse.

"4th. This exact fitting of the two edges to one another could only be effected in one direction; and, if any attempt were made to pull them in the opposite direction, the sides would not collapse, but remained open; from which it appeared, that the action of the needle was confined to separating the fibres of the skin. The knowledge of this fact may perhaps be serviceable in investigating the structure of the skin.

"5th. In any given region of the body, the punctures always affected the same direction.

"6th. In the neck and anterior part of the axilla, they were directed from above downwards.

"7th. On the chest they were parallel to the direction of the ribs, or intercostal spaces.

"8th. In the anterior region of the abdomen they were oblique from above downwards, and seemed to follow the course of the muscular fibres. In the middle, over the linea alba, their direction was from above downwards.

"9th. In the limbs they were always parallel to their axes.

"These facts are not only important with reference to the structure of the skin, but might be exceedingly useful in legal medicine; for instance, in recognizing the possibility of a flat wound having been inflicted by a rounded instrument." (P. 62.)

The conclusions of the Baron, on the effects of punctured wounds of soft parts, are, that they are, generally speaking, without danger, and readily cured, unless the instrument be rusty, (in which case it will act like a file,) or unless it be dipped in some kind of poison, when its ill effects are to be attributed rather to the poison than to the puncture.

*Punctures through the harder substances* are not, however, so free from ill consequences; witness, wounds of the tendon of the biceps in bleeding, which are frequently followed by inflammation, abscess, sloughing of the tendon, and permanent contraction of the limb. The following is a good account of the symptoms produced by such wounds.

"These accidents do not usually occasion any inconvenience till the fourth or fifth day. The patients then complained of deep-seated pains, which are soon accompanied by swelling about the wounded tendon or aponeurosis; then follows a contraction of the limb in the direction of the wound, and incapability of stretching it, at least, without severe pain. Sometimes there is great disturbance of the constitution; fever, attended by disorder of the nervous system, spasms, convulsions, &c. The symptoms may last for weeks, and even for whole months; at the end of which time, the pain and swelling gradually subside, the capability of motion returns, the skin is detached from the fibrous parts beneath, and the parts recover their natural suppleness. These affections, however, do not always terminate in the same fortunate manner: sometimes a chronic abscess forms about the tendon or aponeurosis, which, after it has been opened, spontaneously or by the lancet, remains fistulous, until a slough comes away from the part originally injured. In these cases the adhesions between the fibrous parts and the neighbouring tissues become firmer, and consequently more difficult to destroy; sometimes they last during life, and the cicatrix may be seen to partake in every motion of the tendon to which it is attached." (Pp. 67-68.)

The treatment recommended by the Baron, in these cases, consists in general and local bleeding, emollient cataplasms, and other antiphlogistic remedies. We are indeed surprised that he has omitted to notice the most powerful remedy in our possession, viz. a free division of the aponeurosis. It would be waste of time, were we to endeavour to prove to English surgeons, by the citation of cases, the utility of this proceeding: they have but to enter the walls of any of our hospitals, or turn over the leaves of any periodical, to witness its good effects.

*Wounds of bones* of this order are generally followed by inflammation, abscess between the periosteum and the bone, and necrosis. Baron Dupuytren advocates strongly the early opening of the parts, in order to prevent the spreading of the suppuration.

In the section on *Punctured Wounds of Cavities, and their Contents,* a great difference is shown to exist between such as have, and such as have not muscular, parietes. In the former it requires a very large wound to occasion any effusion of their contents; in the latter, the most trifling puncture is followed by the escape of fluid: the gallbladder will form a good instance of the one, and the intestinal canal of the other. For a further account of the circumstances which influence the effusion or non-effusion of the contents of the latter, we may refer the reader to Mr. Travers' excellent work upon Wounds of the Intestines, where he will find the subject treated at a much greater length than in the work before us. The Baron contents himself with stating, that the distension of the cavity will, of course, render the extravasation of its contents more probable; from whence he derives a caution, which may be perhaps useful in the chapter of accidents, viz. that, should a surgeon be called to a case in which the bladder be wounded, and the instrument remains in the wound, before any attempt be made to extract it, care should be taken to draw off the urine; or, if the heart be wounded, that blood should be previously taken away. Wounds of articular cavities are more liable to be followed by inflammation than those of other parts, and this inflammation rapidly runs down into suppuration, occasioning the necessity for early amputation.

The sum of the Baron's directions, with regard to punctured wounds complicated by the presence of the instruments which inflicted them, is, that the surgeon should extract them as speedily as possible, using force, or even the trepan, if they are fixed in bone, and enlarging the opening, if imbedded in the soft parts. He makes mention, however, of the fact, that foreign bodies may remain enclosed in the flesh without any ill consequences, and relates a most extraordinary case, of an officer who, attempting to commit suicide, pushed a hair-pin into the substance of the heart itself: it did not occasion the slightest derangement of his health, and was only discovered in the post-mortem examination some time afterwards, when he killed himself by a more effectual method. Nevertheless, they are not always so harmless, as is testified by a case of a woman, from whom he extracted above one hundred needles, in different abscesses, and who at last died from exhaustion. Thus we must coincide with the simple yet philosophic opinion of Ambroise Paré, at the termination of the celebrated case of the Duke of Guise, who fought for a considerable time, having the end of a lance buried in his skull, but ultimately recovered: "Donc concluons qu'aucuns meurent de bien petites playes, les autres réchapent de très-grandes, voire qui sont entièrement désespérées, tant aux médecins qu'aux chirurgiens; mais telles choses se doivent quelquefois référer aux températures, et principalement à Dieu qui tient la vie des hommes en sa main." (*Œuvres d'Ambroise Paré*, p. 266, ninth edition, 1633.)

When inflammation and suppuration take place around these foreign bodies, after they have remained tranquil for some time, the wound will be found to become fistulous, and long remain so, unless the cyst which has formed around the extraneous substance be removed.

The section on *Punctured Wounds complicated by the insertion of a Poison* enters at length into the subject, from the sting of the bee to the bite of the viper, the poisoned arrow of the savage, and the virus of rabid animals; but, as it contains nothing remarkably novel, we must pass on to that upon *Punctured Wounds complicated by Disorders of the Nervous System*, such as subsultus, convulsions, pain, and tetanus. After stating that they generally follow injuries of some nerve, and that they are more liable to occur in irritable and plethoric constitutions, he adds this valuable advice: "The influence of moral causes on the production of spasms and convulsions is such, that they are frequently moderated, or even totally prevented, by an affectionate voice or friendly sympathy; hence they should be freely bestowed upon the wounded." (P. 93.) Violent pain not unfrequently follows wounds of the nerves, and lasts for a considerable period, occasioning great distress to the patient, and sometimes disturbance of the health.

Among the means of cure, our author entirely omits the use of blisters, which are sometimes extremely serviceable. Should, however, the pain remain obstinate, excision of a portion of the nerve is commonly practised: this, however, sometimes fails in affording permanent relief, and our author believes the failure to depend on a communication being kept up with the brain by means of the anastomosis of the lateral branches. To obviate this, he recommends the division of the nerve both above and below the injured part, and this he has always found to be attended with success.

On the subject of *Tetanus* he has little to add to the aphorism of Hippocrates: Ὁκοσοι ὑπο τετανου ἁλισκονται, ἐν τεσσαρσιν ἡμερῃσιν ἀπολλυνται. ἐαν δε ταυτας διαφυγωσιν, ὑγιεες γινονται. (Sect. 5. Aph. 6;) or, as it is rendered in the terms of the present day, chronic tetanus may occasionally do well, but the acute is always fatal. Dupuytren, however, lays down the only sensible plan of practice to be pursued, viz. that an endeavour should be made to discover which nerve may have been injured, and that it should be freely divided, prior to the exhibition of any other remedies. We know that, in horses, this plan is constantly successful, and we have been informed, from excellent authority, that the majority of horses affected with traumatic tetanus recover after the division of the injured nerve, and the action of a strong purgative and sweat. The practice has fallen into disuse in this country, because no success worth mentioning has yet attended it: yet surely we should endeavour to remove the cause of the disease before we can expect our remedies to be efficacious.

The last section on punctured wounds, is devoted to the various kinds of inflammation which may be produced by them, and its effects. These, however, are hastily treated, as scarcely forming part of the subject. When speaking of erysipelas, Dupuytren does not fail to advocate the practice of applying a blister in the midst of the affected part, a practice which we have seen eminently successful in his hands, though rejected in this country, under a fear (which we believe to be groundless,) of its producing ill-conditioned ulceration. In circumscribed inflammation and abscess, he advises that they should be left to open spontaneously, unless the process be particularly slow. We must confess that this does not accord with our own experience: the skin becomes diseased before it ulcerates, and is afterwards extremely slow in recovering itself, so as to permit the cicatrization of the sore. In diffuse inflammation he employs free incisions, as the best remedy to prevent suppuration, and as the best plan after it has taken place.

Prior to entering on the details of the various kinds of *Incised Wounds,* our author describes their general characteristics. His observations here, as everywhere else, are excellent, but for the most part too elementary for quotation.

Unwilling as French surgeons in general are to adopt what they term the English method of union by the first intention, Dupuytren is not so bigotted as to deny the excellence of the plan in a cleanly incised wound, (though he sacredly makes the reservation of amputations,) and he dwells at considerable length on the various means of keeping the parts in contact. His directions on this subject are so clear, and at the same time so minute, that they must place his fame as a teacher on a par with his reputation as a surgeon. The point of first importance is the position of the part.

"The position should be such, that the muscles and other parts should be perfectly relaxed; that is to say, the part should be in the state which would be the result of the strongest possible contraction of the injured muscles, or, in other words, their points of origin should approach as close as possible their points of insertion; thus, for example, when the flexor muscles are wounded, the limb should be strongly bent, and, when the extensors, it should be kept extended to the utmost; so in the adductors and abductors. When the wounds are oblique, the parts should be placed in such a midway position as will fulfil, as far as possible, the above indication. There are, however, some parts in which position cannot be rendered available; such as wounds of the head, nose, ears, back, &c. Our whole dependence must then be placed on other methods of bringing the parts into contact. Perfect repose of the body, and of the wounded part especially, is indispensably necessary to favour the cure of wounds by the first intention; the repose of the body can only be obtained by the courage and patience of the patient; the immobility of the injured part may be secured by a variety of mechanical contrivances; such as plasters, bandages, sutures, instruments, &c." (Pp. 151-152.)

It must be confessed, by all who peruse this work, that, if Dupuytren is opposed to union by the first intention, his objections do not arise from his incapacity to employ properly the means required for it. We know not where we can find such good directions for dressing wounds as are contained in the following passage. After enumerating the various kinds of adhesive plasters, and giving the preference to our court plaster, he proceeds thus:

"These different plasters are employed in the form of narrow strips, and varying in number and length according to the size and situation of the wound: when small, one strip will suffice; when large, two, three, or more, are required. As the plaster readily becomes hard, it must be softened previously to its application, either by heat, as in the adhesive plaster, or by moistening it, as in court plaster. The wound should be carefully cleaned, and the parts in the neighbourhood, if there be any hairs on them, should be shaved and well dried; an assistant should then draw the edges of the wound neatly together, and hold them in contact; the surgeon should then apply one end of the strip on one side of the wound, and then carrying the plaster over the edges, so as to hold them together, attach the other end to the opposite side of the wound. If it be requisite to apply more than one strip, a small interval should be left between each, in order to permit the exit of pus, should suppuration take place. When it is a flap-wound, the first strip should be applied from the base to the summit; in other cases, it should be placed across the part where there is the widest separation of the edges. After having applied all the requisite strips, if any one of them should be loose, let one of the edges be raised and drawn sufficiently tight, and reapplied. It is one of the great advantages of these strips that they can easily be tightened, according to the degree of force necessary to bring the edges of the wound in contact. Whatever may be the form of the adhesive plaster, when it is taken off, either to be changed, or because the wound is cured, one end should be raised first; drawing it gently towards the wound, near to which it should be left attached, care being taken to hold the parts together by the finger; the same should be done on the other side, and afterwards the central part should be pulled gradually away in the direction of the wound. If these directions be not attended to, but the plaster be dragged off from one end to the other, a great risk is run of tearing open the cicatrix, as yet soft and recent. These adhesive strips can act only on the skin, the subcutaneous cellular tissue, and those muscles which are attached to the skin; they are unable, therefore, to counterbalance the action of muscles endowed with any force. Hence they are serviceable only in combination with position, and in cases of superficial wounds." (Pp. 153-155.)

His directions as to the application of bandages are equally excellent; but, as the above quotation will furnish our readers with a specimen of his method of treating these subjects, we must pass over the section altogether, and refer those who wish to study them to the work itself.

On the subject of *sutures*, Dupuytren's observations are extremely copious; for he not only describes those ordinarily in use, as the interrupted, the twisted, the glover's, and the quilled sutures, but a variety of others, amongst which are the arched suture (*suture à anse*), and a figure of eight suture (*suture en huit chiffres ou entrecroisée*), both of which are applicable to superficial wounds; others, too, by which the cut surfaces are not brought into contact, which are to be employed only in wounds of the intestines. These again differ from one another as they are calculated to bring the serous surfaces into contact, or, on the other hand, by inserting one end of the intestine into the other, applying the external serous to the internal mucous coat. The latter species of suture occupies a great deal of our author's attention, as he seems to think it the most efficacious means of preventing extravasation: we rather suspect, however, from the results of late experiments in this country, that the plan, though plausible, is impracticable.

We shall therefore leave M. Lembert, M. Jobert, Rhandor, and other writers quoted by the Baron, as authors of the various modifications of these sutures, to such as are curious on the subject, and proceed to the section on the *Union of Wounds after Suppuration*. It contains a most excellent history of the symptoms of the formation of pus after a wound, a description of the various kinds of pus, directions as to the method of dressing wounds which are likely to suppurate, and an account of the different dressings applicable to such cases. We can, however, only afford space to extract the following passage, with which the section concludes.

"The cure of wounds after suppuration may take place in two ways: 1st, by drawing the edges of the wound together; and, 2d, by the formation of a new tissue, which is termed a cicatrix.

"The cure by drawing the edges of the wound together is principally accomplished by a natural effort of the soft parts, which have a tendency to contract from the circumference towards the centre. Art may either assist or oppose this effort, by the position in which it places the wounded parts; the former is effected by placing the edge in contact, the latter, by separating them as widely as possible.

"The edges of the wound should be drawn together whenever their union in that position will not inconvenience the motions of the parts. On the other hand, cicatrization by means of newly-formed substance should be attempted, whenever the other method would give rise to the existence of bridles, contractions, or other impediments to the action of the parts, such as constantly occur after severe burns.

"The former of these methods is much more speedy than the latter, and it should therefore be preferred whenever it is practicable; and it may be looked upon as practicable whenever there is no loss of substance. But, where some part is destroyed, it should be the aim of the surgeon to restore it by encouraging the formation of a new substance. Nevertheless, we must depart from this rule whenever the patient's life may be endangered by the prolongation of the effort requisite for a cure by cicatrization; this precaution is frequently necessary in cases of very extensive burns, and other large wounds, in which it is better that the patients should recover, either weakened or deformed, than die from exhaustion produced by abundant suppuration.

"The cicatrix, even when complete, ought to be the object of much solicitude. As it is at first delicate, and possesses but a very feeble power of resistance, it should be protected from all traction or friction, from the irritating action of the sun or air, from that of greasy substances which are liable to become rancid, and from the relaxation occasioned by baths, &c. This may be most effectually done by covering the parts with compresses of fine linen rag, and surrounding them with a tolerably firm bandage. Sometimes socks made of leather, convex or concave pads, and other apparatus, are required for the purpose. When it is likely to give way, from the constant efforts of the surrounding parts to disunite, it may be secured by means of metallic plates fitted for the purpose.

"But, in spite of all that can be done, it sometimes happens that a recent cicatrix is attacked by an ulcerous inflammation, which will destroy a part, or even the whole of it, with frightful rapidity: our first effort, therefore, must be to prevent it; our next, to arrest it when it does occur; and lastly, to repair the mischief, by the formation of a new cicatrix. These ulcerations are generally superficial, and embrace only the surface of the new cutaneous tissue that forms the cicatrix.

"It might appear that the ends of both nature and art are attained immediately that a wound is covered by its cicatrix; but it often happens that this cicatrix is badly formed, that it causes the contraction or obliteration of certain openings, or that it has enlarged others to an unnatural extent, or that it has occasioned bridles, contractions, and other deformities. Here then a new path opens to the surgeon: in order to obtain a cure exempt from such inconveniences, he must commence by destroying all that has been done. He must divide and lay open the cicatrix, separate the edges of the wound, and perform other operations having for their object the conversion of parts into a recent wound, which may again be healed in any convenient manner.

"But we should never determine to destroy a labour thus completed, without having carefully weighed the advantages and disadvantages of the operation. In all cases we must take care not to compromise the life of the patient, in order to obtain some second-rate or uncertain advantage, and never to lose sight of the fact, that small operations, in certain states of the constitution, may prove as fatal as the most severe."

In the section on *Wounds traversing Parts of different degrees of Vitality,* Dupuytren recommends an endeavour to unite the parts by the first intention in all cases, though he advises the surgeon to be ready to break up the adhesions, should any symptoms of suppuration supervene.

We must omit the sections on *Flap Wounds,* and on the *Union of Parts completely separated from the Body.* While treating of the former, he speaks of the rhinoplastic operation, and upon the latter subject he relates some remarkable instances of union after the lapse of a considerable time, such as an hour or two.

The chapter upon *Wounds by Cutting and Puncturing Instruments* is short, but contains some good hints upon the subject of internal hemorrhage. The Baron relates a case of hemorrhage from one of the intercostal arteries into the pleura, where the wound had been injudiciously closed; and he would recommend in such cases its enlargement, and that the artery should be tied.

He mentions also the frequency of recovery after severe wounds of the abdomen, and attributes this to the sword pushing aside the viscera, instead of wounding them. This, however, since the researches of Mr. Travers, is a disputed point. We should have been glad if the Baron, taking the hint from his own experiments with the needle, had directed some of his young fencing friends to try on the dead subject, and this important point might then have been set at rest. In speaking of the means of cure, he mentions the plans of suction and compression: the latter he would avoid, on account of the pain inflicted by it, when inflammation follows the wound. He equally disapproves of suction, especially in cases where any large vessel has been wounded, as it causes the constant removal of the clot, upon the formation of which the patient's life depends.

Though our author objects so strongly to the plan of suction, we trust our readers are not of the same opinion, as we intend to put it in full force upon the chapter on *Lacerated Wounds;* a class of accidents which, since the increased employment of machinery, are daily becoming more frequent, and against which every surgeon should be perfectly provided.

He divides the lacerated wounds into three species: 1st, the ordinary lacerated wound by a blunt instrument; 2d, that produced by the rupture of a viscus; and 3d, that occasioned by a violent wrench, as when a limb is dragged off from the body: as however these injuries strongly resemble one another, they are classed together in the same chapter. The ordinary lacerated wound may be caused by the violent withdrawal of puncturing or hooked instruments, as the bayonet or halberd in military surgery, and the carpenter's saw, or the horns of animals, in civil practice. One of the most frequent methods of its production is by the protrusion of the bone in compound fractures. The ruptured wound is generally the consequence of overdistension, or the weakness of the parietes of some hollow viscus; a tendon, however, is not unfrequently broken through by the action of its muscle, and an aneurismal pouch from the diseased state of its coats. The ruptures are sometimes produced by external force; thus, a blow on the epigastrium, after a tolerably full meal, will sometimes occasion rupture of the stomach. When a viscus of the importance even of the stomach or bladder is suddenly ruptured by a blow, or by distension, it is not, as might have been supposed, followed by immediate death: the patient may survive for several days, and then die of acute inflammation, the consequence of the extravasation. Rupture of the intestinal canal will sometimes happen, either from distension with wind, or from their sudden and violent contraction, or when suffering from disease and thickening of their coats; but it must be remembered that extravasation of the stercoraceous matter does not necessarily occur in these cases, and the patients may recover, adhesions having formed between the ruptured parts and the viscera in contact with them. Lacerated wounds may exist in two states, not only different in themselves, but in their consequences; they may occur either in the external parts in contact with the air, or in the internal parts excluded from it: in the former case they always suppurate, in the latter this process is extremely rare.

Lacerated wounds have generally a very uneven surface, arising from the different degrees of resistance offered by the several parts. The edges are slightly ecchymosed, and there is rarely any violent hemorrhage; this is, however, more common in internal ruptured wounds than in those which are external. When it takes place in a parenchymatous viscus, a pouch is formed, in which the blood coagulates and becomes absorbed; in most cases, without any serious consequences.

Should hemorrhage occur in the external wounds, it must of course be stopped in the ordinary manner. When this has been done, the surgeon should draw the edges of the wound together by the means recommended in the chapter on incised wounds. If it be an internal organ that is ruptured, as the stomach, the only means in our power of retaining the edges together is to keep it constantly empty; the other indication is to moderate the inflammation consequent on the injury. When patients recover from these accidents, it is generally by the formation of adhesions, as before mentioned; sometimes, however, abscesses form, and the matter comes away from them tinged with the secretion or contents of the viscus. The cicatrices are seldom well formed, and various inconveniences may result from them; such as weakness of the affected muscle, hernia when the wound is in the parietes of the abdomen, &c.

When a limb is torn from the body, it is seldom the effect of direct force; in most instances, this is combined with torsion, as, for instance, in the almost exploded practice of accoucheurs twisting off children's arms. Our author relates several cases of this species of accident, in which arms and legs were taken off by the motion of a wheel, and in all of which the patients recovered. The principal characters which distinguish these wounds are the extreme inequality of the surface, (the projection being principally on the side of the limb, and the depressions on the stump,) and the nearly constant absence of hemorrhage. This arises partly from the retraction of the vessels, and partly from their structure. As the three coats of the arteries possess different degrees of tenacity, the inner one healing first, then the middle, and lastly, the cellular coat, the end of the vessel presents the form of a cone with its apex turned outwards; in this cone a clot is formed, and inflammation speedily takes place, which obliterates the canal. These accidents are less fatal than might have been supposed; there is but little pain, and no hemorrhage, and therefore the cure depends but little upon art. Should any hemorrhage take place, the usual means must be had recourse to. The surgeon must likewise pay some attention to the surface of the wound, and, should any parts project very much, he must remove them by the knife. The edges of the wound must be drawn together, and, should any unpleasant symptom arise, it must be met as in other cases.

The Baron prefaces his observations upon *Contused Wounds* by some remarks on concussion and coma. But, as these are not treated at length, we shall omit them, and refer our readers to English works, where they may find them more minutely discussed. He divides contusions, according to their severity, into four classes. The "contusion au premier degrê" answers to the ordinary bruise; the second embraces those cases where pouches full of blood are formed by the rupture of a vessel of tolerable size; the third may or may not be accompanied by a lacerated wound, and is generally followed by the death of the part; and the fourth occasions instant disorganization.

Cases very similar to these last, viz. *Crushed Wounds,* are treated of in the following chapter; and we are thus gradually led to the consideration of *Gunshot Wounds,* the subject which is usually supposed to be of most interest to military surgeons, but on which we shall not dwell very long, as we possess most excellent treatises upon the subject in our own language.

The Baron commences by some remarks on the *Physical Effects of Projectiles from Firearms,* the principal of which are as follows. When the gun has been discharged close to the wounded part, the opening by which the ball enters is smaller than that by which it makes its exit; but if at a distance, so that the ball is nearly spent, then the reverse will be observed. In the one case the powder will adhere to the wound, in the other, none will be found. The canal made by the ball in the former instance will be conical: hence the extreme difficulty of extracting it, when lodged in a bone, without the use of a trephine. In passing through a flat or spongy bone, it makes a clean hole, without splinters; but, in perforating a long bone, the wound is generally ragged, with splinters of bone sticking into the soft parts; at other times it fractures these bones, without further injury. When a ball strikes a convex part of the body obliquely, it not unfrequently takes a circuitous course, and escapes at a point opposite to its entrance: thus, it may strike the sternum, and make its exit near the spinous processes of the vertebræ, without perforating the cavity of the chest; so in the head, &c. If linen be loosely applied to the body, the ball will frequently push a large portion, like a funnel, before it, into the wound; but, if the linen be tightly strained, it will carry away only a piece of its own size. It is not an uncommon accident for the ball to be broken by striking against a bone, and for the two fragments to go in different directions; it may otherwise alter in shape, so as to render its extraction extremely difficult. Such are the chief physical effects of these projectiles themselves; but they may be complicated, by means of splinters of wood, portions of the dress or arms, driven before them. These complications, however, vary infinitely; and we have not space to follow the author through his disquisitions upon their different consequences. We must likewise pass over the *Vital Effects of Gunshot Wounds;* and shall conclude this article by abridging

Dupuytren's opinions with regard to *Amputation in Cases of Gunshot Wounds.*

A simple wound of the soft parts seldom requires the loss of the limb; only in cases where, from constitutional or other causes, traumatic gangrene is about to take place. Fractures of bones are the most common wounds requiring amputation, but here the surgeon must exercise some discretion. If the bone be not much shattered, and if the soft parts are not much bruised, and the constitution is tolerably tranquil, the patient, being treated as with a severe compound fracture, may perhaps retain his limb, especially if he be extremely young; but, if these favourable circumstances do not exist, recourse should be had to amputation. Of course, the chances are more in favour of a patient who is admitted at once into a civil hospital, than of him who is wounded and treated on a field of battle.

Wounds of the large joints, or vessels, if they cannot be secured, require the removal of the limb; as also very extensive lacerations of the soft parts, by the explosion of mortars, &c. Another circumstance must also be taken into consideration, viz. whether the patient has sufficient strength to bear up against a tedious suppuration; if not, this is sufficient reason for the operation.

As to the period at which the amputation should be performed, Dupuytren agrees with all reasonable surgeons, that it is more successful when done within six or eight hours, than after inflammation has commenced: if, however, it should be delayed beyond that time, the surgeon should not wait till the patient's strength is exhausted; but, as soon as he perceives that his treatment will be unsuccessful in saving the limb, he should lose no time in removing it. The occurrence of hemorrhage or traumatic gangrene would only increase the necessity for instant operation.

Our author, in cases of amputation for gunshot wounds, recommends the attempt to heal the stump by the first intention, but he makes the reservation, that it can never be completely effected. The endeavour will, however, lessen the wound, which is to be healed by the slower process of granulation. He cautions surgeons to remember that the operation, after all precautions, will frequently prove fatal: he says that one patient dies out of six operated upon.

With these observations the Baron finishes the first volume of his work. His second will contain his opinions upon hemorrhage, traumatic fever, visceral abscesses, hospital gangrene, &c., and the nature of wounds in each part of the body.

We now close the book, and shall say but little in its praise at present. This does not arise from any doubt that the second volume will equal the first, but from a conviction that the reputation of Baron Dupuytren will render almost every member of the profession curious to peruse his lectures. When once opened, we may safely leave them without other observation, than to caution the reader not to sacrifice every other engagement to their engrossing interest.

